# An Inter‐ and Intra‐Rater Agreement Assessment of Two Novel Classification Systems for Coronal Imbalance in Adult Scoliosis and Spine Deformity

**DOI:** 10.1111/os.14356

**Published:** 2025-01-20

**Authors:** Muradil Mardan, Mardan Mamat, Parhat Yasin, Xiao‐Yu Cai, Guo‐Jun Fan, Tao Xu, Bo Li, Peng‐Bo Chen, Ze‐Yu Lu, Wei‐Bin Sheng, Sheng‐Dan Jiang, Lei‐Sheng Jiang, Xin‐Feng Zheng

**Affiliations:** ^1^ Department of Spine Center Xinhua Hospital Affiliated to Shanghai Jiaotong University School of Medicine Shanghai China; ^2^ Department of Spine Surgery The First Affiliated Hospital of Xinjiang Medical University Urumqi China; ^3^ Department of Orthopedics Urumqi First People's Hospital Urumqi China

**Keywords:** adult spinal deformity, agreement study, coronal malalignment, reliability, reproducibility

## Abstract

**Objective:**

Coronal malalignment is a common feature of adult spinal deformity, and accurate classification is essential for diagnosis and treatment planning. However, variations in interpretation among clinicians can impact classification consistency. By assessing the reliability and applicability of these systems across different medical experts, this study seeks to establish a standardized approach to enhance clinical outcomes. This study aimed to evaluate the inter‐ and intra‐observer agreement of two classification systems for coronal malalignment in adult spinal deformity patients, as proposed by Qiu et al. and Obeid et al.

**Methods:**

We analyzed 70 cases of adult spinal deformity collected between January 1, 2010, and April 20, 2023, using the classification systems proposed by Qiu et al. and Obeid et al. To assess inter‐ and intra‐rater agreement, the same group of researchers re‐evaluated all cases in a random order after a 4‐week interval. We used the kappa statistic (*κ*) for inter‐ and intra‐rater agreement assessment.

**Results:**

Qiu's classification system: Inter‐rater agreement: Substantial agreement (*κ* = 0.76; 95% CI: 0.72–0.80) for Type A, Type B, and Type C. Intra‐rater agreement: Nearly perfect agreement (*κ* = 0.83; 95% CI: 0.78–0.89) within raters for Type A, Type B, and Type C.

Obeid's classification system: Inter‐rater agreement: Almost perfect agreement (*κ* = 0.85; 95% CI: 0.83–0.87) for Type 0, Type 1, and Type 2. Complete system: Substantial agreement (*κ* = 0.68; 95% CI: 0.65–0.71) for all types and subtypes. Intra‐rater agreement: Almost perfect at the type level (*κ* = 0.88; 95% CI: 0.83–0.93) and substantial at the subtype level (*κ* = 0.75; 95% CI: 0.65–0.85).

**Conclusions:**

The research findings indicate a high level of agreement between the classification system described by Qiu et al. and the classification system proposed by Obeid et al. This agreement supports the widespread adoption and utilization of these classification systems in future clinical studies.

## Graphical abstract

This study assesses the inter‐ and intra‐rater agreement of two classification systems for coronal imbalance in adult scoliosis, demonstrating their reliability, and potential for widespread clinical application in improving diagnostic accuracy and treatment planning.

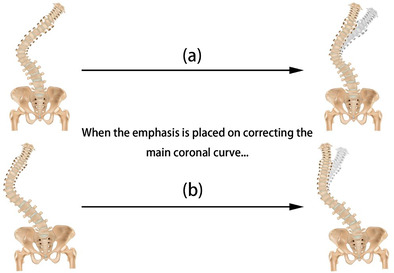

## 1 Introduction

The spine plays a crucial role in the human body, positioned centrally in the trunk, connecting the skull above with the pelvis below. It not only supports body weight through its complex system of bones, ligaments, muscles, and joint capsules but also facilitates movement. Typically, individuals adjust their posture to align the center of gravity with the vertical midline between their feet, minimizing the energy required for balance. However, when external factors disrupt this equilibrium, the body activates compensatory mechanisms, which often increase energy demand and place strain on muscles and other tissues, leading to discomfort and pain [1]. Normally, the body instinctively adjusts spinal positioning to maintain balance [2, 3]. Spinal balance can be categorized into three aspects: coronal, sagittal, and axial balance, and when external forces exceed the spine's compensatory capacity, imbalance in the corresponding aspect may occur [4]. Scholars have extensively researched imbalances caused by external factors [5, 6, 7, 8, 9, 10], with particular focus on the classification and treatment of coronal malalignment (CM).

**FIGURE 1:**
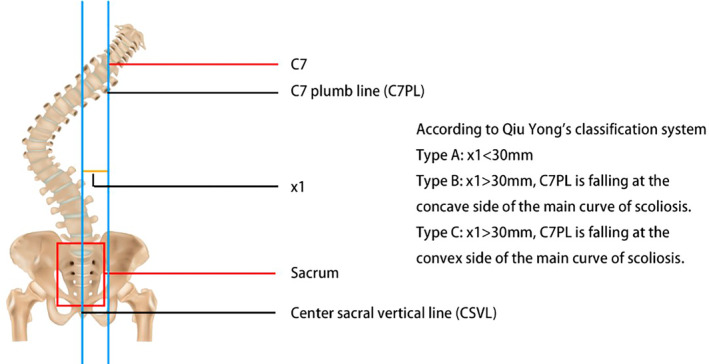
Classification of coronal malalignment according to Qiu et al.

**FIGURE 2:**
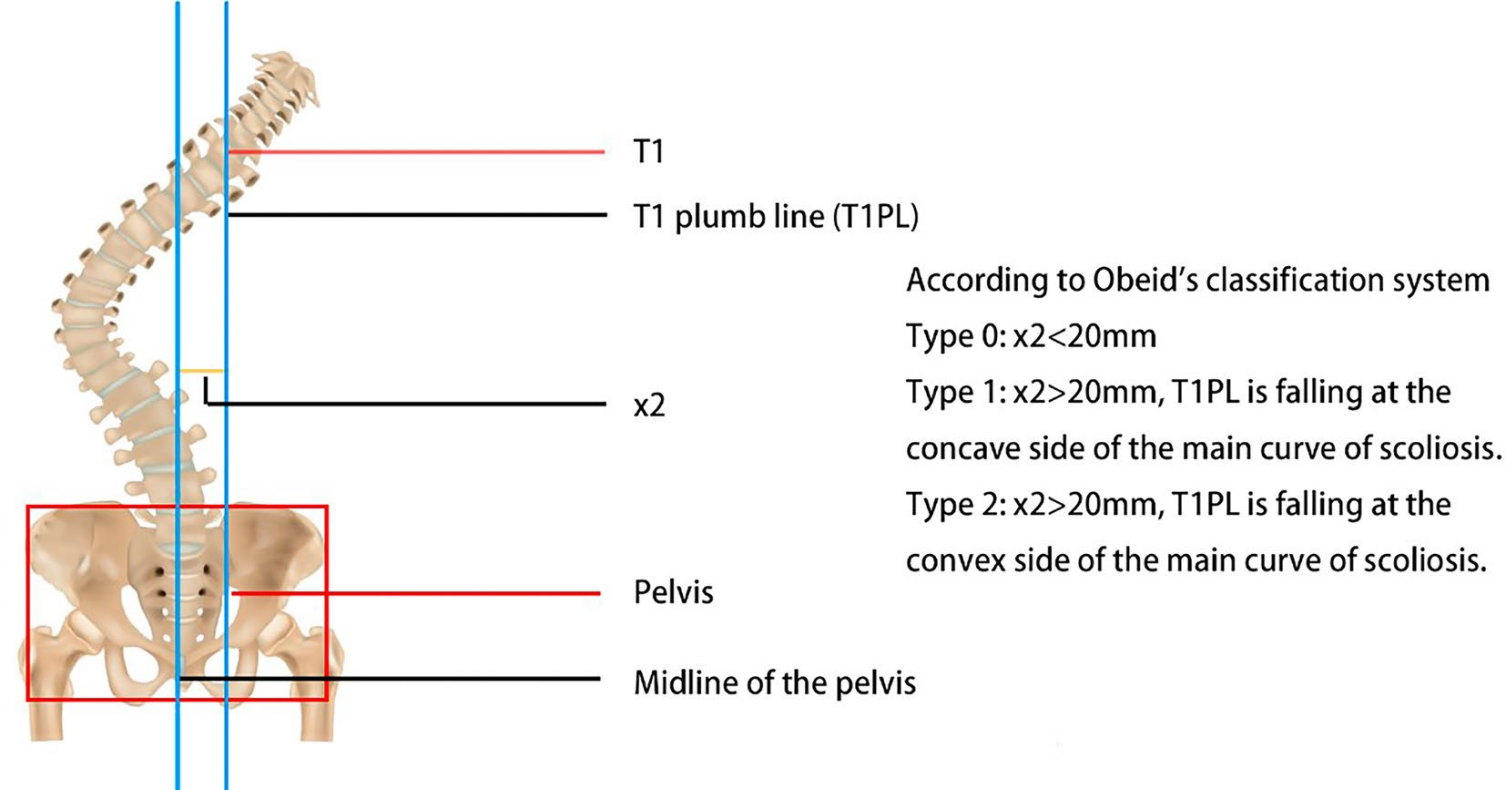
Classification of coronal malalignment according to Obeid et al.

In 2009, Qiu et al. [11] introduced a classification system for degenerative lumbar scoliosis based on the distance between the C7 plumb line and the center sacral vertical line, comprising three types (Table 1 and Figure 1). The primary aim of this classification was to address the variation in outcomes observed when correcting the main scoliosis curve in different patients: in concave CM cases, correcting the main curve often reduces CM, whereas, in convex CM, correction may increase CM. After Bao et al. [12] introduced this method in 2016, it gained widespread acceptance, and some researchers even referred to it as the “Bao classification” [13, 14]. In 2019, Obeid et al. [15] proposed a new classification for adult spinal deformity (ASD) focused on CM. They defined CM as a lateral deviation of the T1 plumb line of more than 20 mm from the midline of the pelvis, allowing for surgical strategy adjustments based on patient‐specific characteristics to improve outcomes. Their classification considers multiple factors, including the distance between T1PL and the central pelvic line, the stiffness of the main coronal curve, and the mobility and degeneration of the lumbosacral junction. This system consists of three major types and six subtypes (Table 2 and Figure 2).

**TABLE 1:** Classification of coronal malalignment according to Qiu et al.

Type	Description
Type A	The distance between C7PL and CSVL is less than 30 mm
Type B	C7PL is falling at the concave side of the main curve of scoliosis
Type C	C7PL is falling at the convex side of the main curve of scoliosis

**TABLE 2:** Classification of coronal malalignment according to Obeid et al.

Type	Description
Type 0	Lateral deviation of the T1PL with substantial displacement from the midline of the pelvis for less than 20 mm
Type 1	CM^a^ with coronal T1PL falling at the side of the concavity of the main coronal curve
1A	Main curve with apex between T12 and L4
1A1	Main curve is flexible on bending or potentially after posterior release
1A2	Main curve is very rigid or fused
1B	Main curve with apex above T12
Type 2	CM with coronal T1PL falling at the side of the convexity of the main coronal curve
2A	Main curve with apex between T12 and L4
2A1	L4–S1 not degenerated and coronally mobile
2A2	L4–S1 degenerated or stiff
2B	Main curve with apex below L4^b^

^a^ Lateral deviation of the T1PL with substantial displacement from the midline of the pelvis for more than 20 mm.

^b^ This is a convex‐like CM as it is apparently a convex CM in respect to the L curve, yet in theory, it should be a concave CM in respect to the LS curve (main curve in this case).

Both Qiu and Obeid classification systems were selected for this study due to their focus on CM in ASD and their relevance in clinical settings. These systems complement other established classifications, such as the Lenke classification, which is primarily applied to adolescent idiopathic scoliosis (AIS). The Qiu classification system is widely recognized for its simplicity and ease of application in cases of degenerative scoliosis. In contrast, the Obeid classification system provides a more detailed framework, accounting for coronal mobility and lumbosacral degeneration, making it particularly suitable for complex deformity cases. While the Lenke classification emphasizes sagittal and coronal balance, it is less specific for ASD and does not directly address CM. Therefore, the Qiu and Obeid classification systems were chosen as they more directly address coronal imbalance, catering to the unique characteristics of adult scoliosis.

A reliable classification system is essential for developing effective treatment strategies and delivering precise medical care. A comprehensive classification system enables healthcare professionals to better understand patients' conditions and tailor treatments accordingly, thus enhancing healthcare quality and improving patients' quality of life. However, developing a disease classification system requires multiple rounds of intra‐ and inter‐observer consistency testing to verify its reliability. The purposes of this study are to: (i) evaluate the inter‐observer agreement of the Qiu and Obeid classification systems for CM in ASD cases; (ii) assess the intra‐observer consistency of these systems over time; and (iii) investigate factors affecting agreement, with a focus on differences in measurement criteria and threshold sensitivity between the two systems.

## 2 Material and Methods

### 2.1 Sample Size Estimation

Sample size estimation was performed using R. We employed a confidence interval (CI) approach for determining the sample size in inter‐observer agreement studies involving multiple raters, as described by Rotondi et al. [16]. Using a lower limit of 0.71 and an upper limit of 0.90 (an expected substantial agreement) for six evaluators and a 95% CI, we calculated the required sample size to be 62 cases.

### 2.2 Patient Selection and Inclusion/Exclusion Criteria

This study included adult patients with spinal deformities admitted to the First Affiliated Hospital of Xinjiang Medical University and Xinhua Hospital Affiliated to Shanghai Jiao Tong University between January 1, 2010, and April 20, 2023. All patients were required to undergo standing position X‐rays of the whole spine in both coronal and sagittal planes, both before and after surgery. Additionally, X‐rays of the coronal plane of the spine in left and right sidebending were obtained prior to the operation.

Inclusion criteria:

1. Age over 18 years.

2. Coronal scoliosis with Cobb angle greater than 10°.

3. Availability of complete imaging data (standing full‐spine anteroposterior X‐ray, standing full‐spine lateral X‐ray, and full‐spine bending X‐ray images.).

Exclusion criteria:

1. Adult stage of AIS.

2. Patients with spinal tumor, tuberculosis, spondylolisthesis, history of pelvic trauma, history of spinal surgery, history of hip, and knee surgery.

3. Lower limb length difference greater than 2.0 cm.

4. Non‐structural kyphosis.

We obtained institutional review board approval to conduct this study. Informed consent was waived since it was a retrospective study with no interventions or direct contact with the patients.

### 2.3 Imaging Acquisition and Rater Training

After downloading Surgimap 2.3.2 from the official Surgimap website, the X‐ray image data was imported and measured.

The cases were selected by an independent researcher who did not participate in the classification stage of this study. Cases were chosen from a large hospital database and included patients with all types and subtypes of ASD as defined by Qiu et al. and Obeid et al. Six spinal surgeons from our hospital participated in the study. All of these surgeons were experienced attending physicians with a minimum of 1 year of experience in spinal diagnosis and treatment. They received training in operating the Surgimap software. After completing the training, the X‐rays of the patients were independently measured by each of the six physicians. Prior to the assessment, the physicians also underwent training in the classification system to resolve any uncertainties and ensure a standardized evaluation process. The evaluators blinded the patients’ basic characteristics, such as age, sex, body mass index (BMI), and comorbidities, to avoid any influence on the classification process.

### 2.4 Measurement and Classification

**FIGURE 3:**
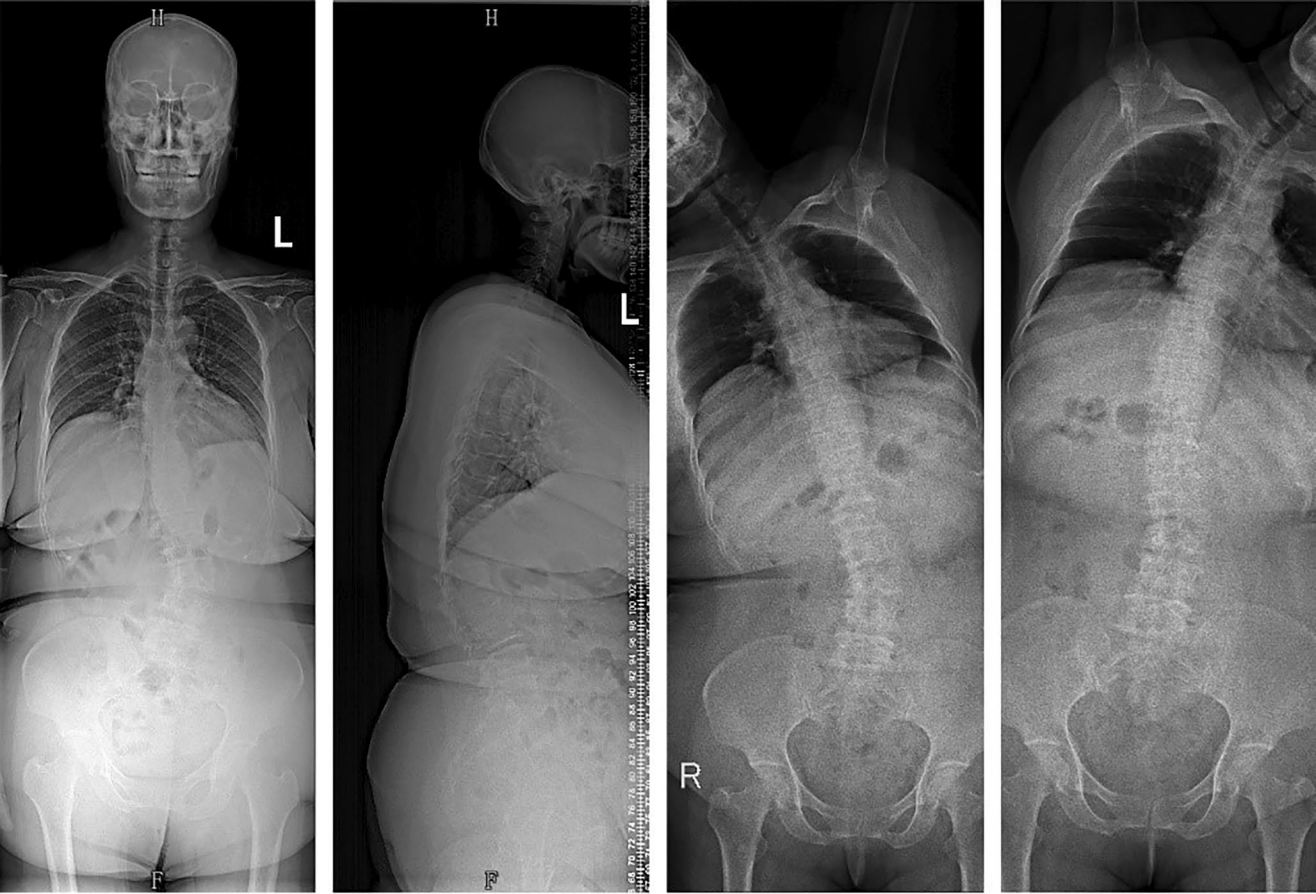
A case of ASD without CM. The Cobb angle of the main spinal curve in this patient is 33.4°. The horizontal distance between C7PL and CSVL is 6.1 mm, and the horizontal distance between T1PL and the midline of the pelvis is 5.5 mm. According to Qiu's classification criteria, it is classified as Type A, and according to Obeid's classification criteria, it is classified as Type 0.

**FIGURE 4:**
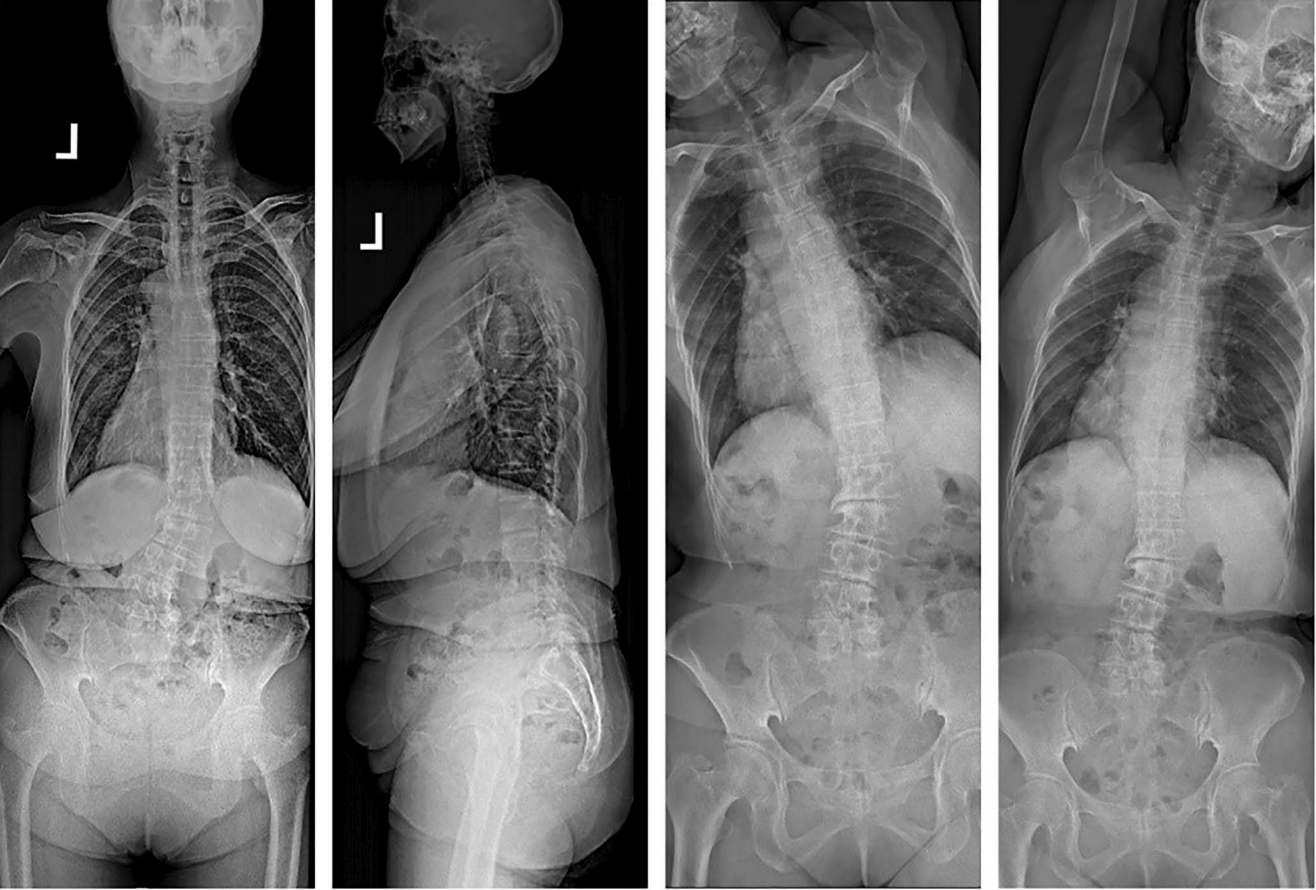
A case of concave CM in ASD. The Cobb angle of the main spinal curve in this patient is 35.1°. The horizontal distance between C7PL and CSVL is 45.8 mm, and the horizontal distance between T1PL and the midline of the pelvis is 44.9 mm. Additionally, both C7PL and T1PL fall on the concave side of the main spinal curve. According to Qiu's classification criteria, it is classified as Type B, and according to Obeid's classification criteria, it is classified as Type 1.

**FIGURE 5:**
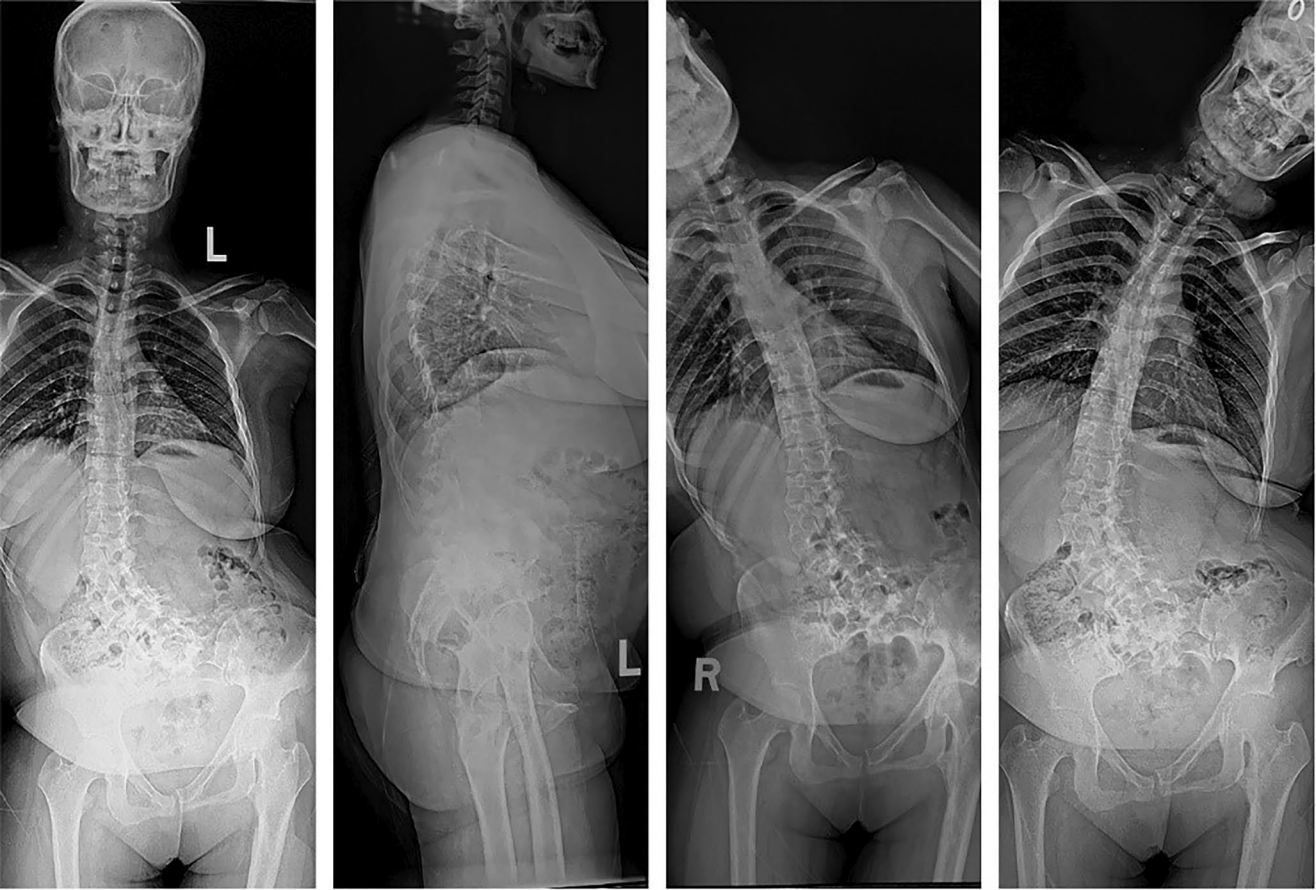
A case of convex CM in ASD. The Cobb angle of the main spinal curve in this patient is 57.0°. The horizontal distance between C7PL and CSVL is 85.2 mm, and the horizontal distance between T1PL and the midline of the pelvis is 88.4 mm. Additionally, both C7PL and T1PL fall on the convex side of the main spinal curve. According to Qiu's classification criteria, it is classified as Type C, and according to Obeid's classification criteria, it is classified as Type 2.

Each physician measured the distance between C7PL and CSVL, observed the horizontal deviation from T1PL to the midline of the pelvis, and assessed the stiffness of the primary curve, coronal mobility at the lumbosacral junction, and the presence of degeneration. CSVL was defined as a vertical line through the center of S1, while C7PL was determined by a line through the midpoint of C7. T1PL was similarly identified using the midpoint of T1. To minimize errors, each X‐ray was calibrated using the known distance between reference markers on the image, and disagreements in measurements were resolved through discussions. These procedures were implemented to ensure consistency and reproducibility across all raters. They then classified the cases according to Qiu's and Obeid's systems, respectively (Figures 3, 4, 5). (Although Qiu et al.'s classification system is intended for classifying degenerative scoliosis‐associated CM, we believe that the reason behind their classification into convex CM and concave CM is primarily due to the different effects observed during the correction of the main scoliotic curve. Based on this, we consider that Qiu et al.'s classification system can be applied to CM caused by non‐degenerative spinal curvature as well. In this study, we also utilized Qiu et al.'s classification system to classify CM associated with non‐degenerative spinal curvature. However, to maintain the consistency of our study population and ensure the applicability of the classification system, we chose to exclude adult‐stage AIS cases. Adult‐stage AIS, which refers to scoliosis originating in adolescence and persisting into adulthood, has a distinct etiology compared to other forms of adult scoliosis. Including adult‐stage AIS could introduce confounding factors and may affect the interpretation of the classification results. Therefore, we excluded these cases to maintain a more homogeneous adult patient cohort.)

### 2.5 Statistical Analysis

SPSS 25.0 statistical software was employed for data processing in this study. Since the classification systems proposed by Qiu et al. [11] and Obeid et al. [15] involved categorical data, the *κ* test was utilized to assess the diagnostic consistency. Inter‐observer agreement was determined by comparing the initial assessments made by all assessors. Intra‐observer agreement was calculated by comparing the assessments made by the same evaluator on two separate occasions for the same group of patients. The two assessments were conducted with a 4‐week interval between them and were presented in a randomized order to minimize the potential impact of recall bias.

In this study, we used the *κ* statistic to evaluate the consistency of raters in classifying cases according to Qiu's and Obeid's classification systems. The *κ* values were calculated to quantify both inter‐ and intra‐rater agreement, providing an overall assessment of the reliability of the classification systems. To ensure the accuracy of the results, we followed a standardized measurement protocol and resolved any disagreements through consensus discussions. The final *κ* values were expressed along with their corresponding 95% CIs to represent the precision of our estimates. Levels of the agreement for *κ* were determined as proposed by Landis et al. [17] Agreement was considered almost perfect if *κ* = 0.81–1.00, substantial if *κ* = 0.61–0.80, moderate if *κ* = 0.41–0.60, fair if *κ* = 0.21–0.40, slight if *κ* = 0–0.20 and poor if *κ* < 0.

## 3 Results

### 3.1 Patient Selection and Exclusion

The study initially included 113 patients with ASD. Subsequently, 15 patients were excluded due to being in the adult stage of AIS. Additionally, nine patients were excluded because they had undergone spinal curvature correction surgery before admission, and 19 patients were excluded due to a lack of imaging data. As a result, the final analysis included 70 patients, comprising of 33 males and 37 females, with an average age of (48.23 ± 11.77) years.

### 3.2 Qiu's Classification System

#### 3.2.1 Inter‐Rater Agreement

The evaluation of the classification categories (Type A, Type B, and Type C) demonstrated a significant level of agreement among raters, with a *κ* value of 0.76 (95% CI: 0.72–0.80).

#### 3.2.2 Intra‐Rater Agreement

The evaluation of the classification categories (Type A, Type B, and Type C) showed a nearly perfect level of agreement within raters, with a κ value of 0.83 (95% CI: 0.78–0.89).

### 3.3 Obeid's Classification System

#### 3.3.1 Inter‐Rater Agreement

When assessing the main classification categories (Type 0, Type 1, and Type 2), there was an almost perfect level of agreement among raters, with a *κ* value of 0.85 (95% CI: 0.83–0.87). The analysis of the complete classification system (including types and subtypes) revealed a substantial level of agreement, with a *κ* value of 0.68 (95% CI: 0.65–0.71).

#### 3.3.2 Intra‐Rater Agreement

At the type level, there was an almost perfect level of agreement within raters (*κ* = 0.88; 95% CI: 0.83–0.93). At the subtype level, the agreement was substantial (*κ* = 0.75; 95% CI: 0.65–0.85).

### 3.4 Summary of Agreement Results

The inter‐ and intra‐rater agreement assessment of Qiu's classification system and Obeid's classification system is presented in Table 3. Further detailed classification results for each patient and rater are provided in File SS1, and a summary of these results are included in File SS2 for a comprehensive overview.

**TABLE 3:** The inter‐ and intra‐rater agreement assessment of Qiu's classification system and Obeid's classification system.

Classification system	Inter‐rater agreement κ (95% CI)	Intra‐rater agreement κ (95% CI)
Classification system of Qiu		
Per category agreement (Type A, Type B, and Type C)	0.76 (0.72–0.80)	0.83 (0.78–0.89)
Classification system of Obeid		
Per category agreement (Type 0, Type 1, and Type 2)	0.85 (0.83–0.87)	0.88 (0.83–0.93)
Per sub‐category agreement (Type 1A1, Type 2A2, etc.)	0.68 (0.65–0.71)	0.75 (0.65–0.85)

## 4 Discussion

### 4.1 Summary of Principal Findings

In this study, we evaluated the inter‐ and intra‐rater agreement of Qiu's and Obeid's classification systems for CM in ASD cases. Our findings suggest that while both systems demonstrate substantial agreement, differences in measurement criteria and threshold sensitivity impact the consistency of classification outcomes. Additionally, factors such as patient positioning and measurement protocol variability contribute to observed discrepancies, highlighting areas for standardization.

### 4.2 Clinical Significance of CM in ASD

**FIGURE 6:**
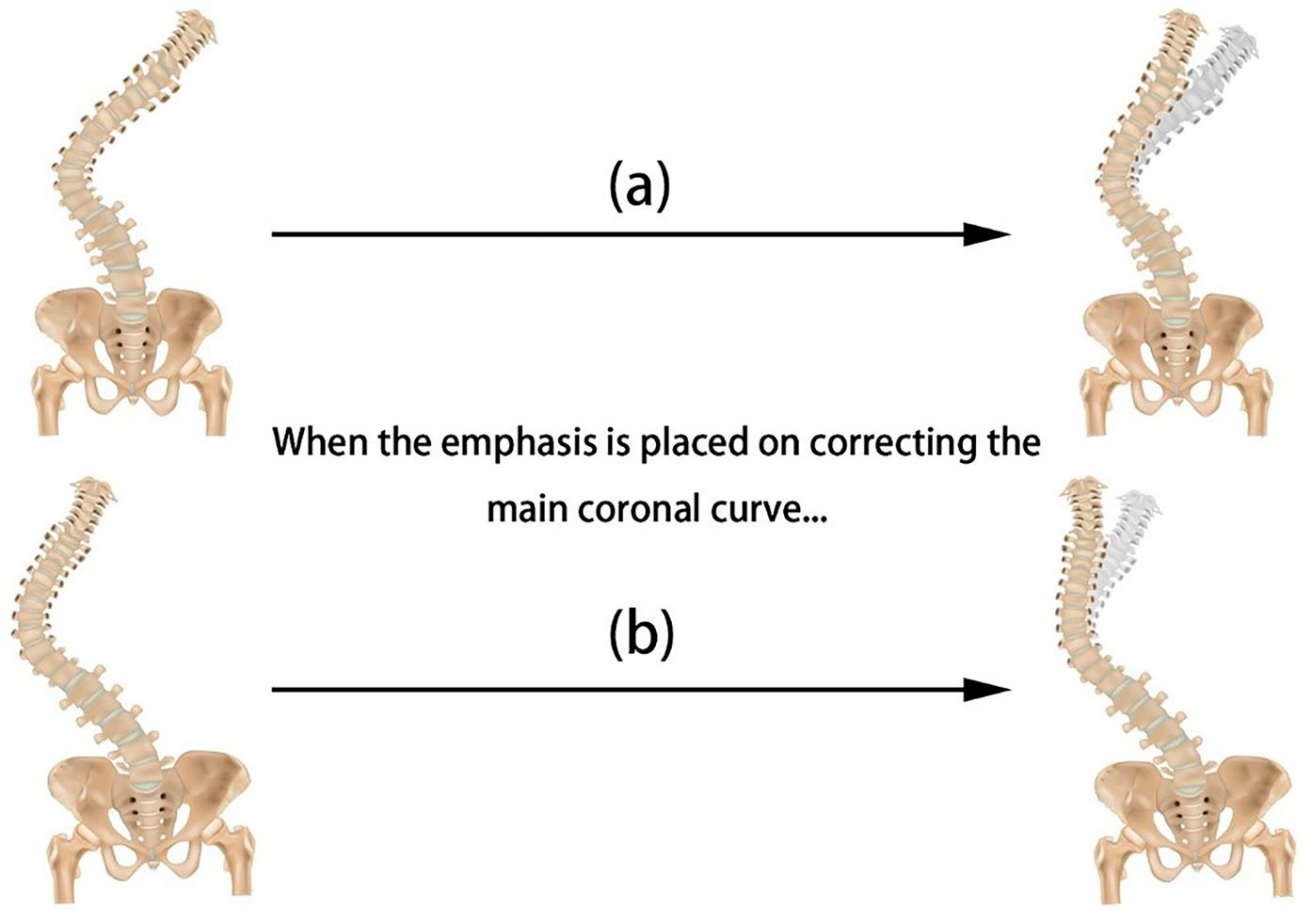
Illustration of CM during correction of the main coronal curve. (a) As shown in the above diagram, when correcting the main curve for patients with concave CM, a reduction in the degree of coronal plane imbalance becomes apparent. (b) As shown in the above diagram, when correcting the main curve for patients with convex CM, an increase in the degree of coronal plane imbalance becomes apparent.

ASD is one of the most disabling conditions, with an incidence rate of 32% in adults and up to 60% in the elderly population [18]. Surgical intervention is considered an effective approach for treating ASD, as it aims to correct the sagittal and coronal imbalances of the spine, thereby reducing patients' pain and improving their quality of life [19, 20]. In the past, the focus of surgical procedures primarily revolved around correcting sagittal plane imbalances. However, in recent times, there has been an increasing emphasis on addressing coronal plane imbalances in treatments. Recent research indicates that CM can lead to pain, disability, and functional impairments [21, 22]. In this context, Qiu et al. divided CM into three major types based on the distance between C7PL and CSVL and the location of the apex of the spinal curve. Subsequently, Obeid et al. classified CM into three major types and six subtypes based on the horizontal distance between the T1PL and the pelvic plumb line, the location of the apex of the spinal curve, the mobility of the main curve, the coronal plane mobility at the lumbosacral junction, and the degeneration at the lumbosacral junction. The relevance of these patterns depends on the various effects that correcting the main coronal curve has on each pattern. In the case of concave CM, correcting the main curve is accompanied by a reduction in CM. Conversely, correcting the main curve in convex CM leads to an increase in CM (Figure 6).

### 4.3 Comparison Between Qiu and Obeid Classification Systems

In this study, we conducted an assessment of inter‐ and intra‐rater agreement for both Qiu's and Obeid's classification systems. Although the two systems share similarities in defining CM, they differ significantly in the selection of measurement landmarks and the criteria for CM diagnosis. Specifically, Qiu's classification measures the distance between C7PL and CSVL, using a threshold of 30 mm to define CM, whereas Obeid's classification uses the distance between T1PL and the pelvic plumb line, with a lower threshold of 20 mm. These differences can lead to variability in classification outcomes, particularly for cases where the measured distance is near these critical thresholds.

### 4.4 Influence of Measurement Thresholds on Observer Consistency

**FIGURE 7:**
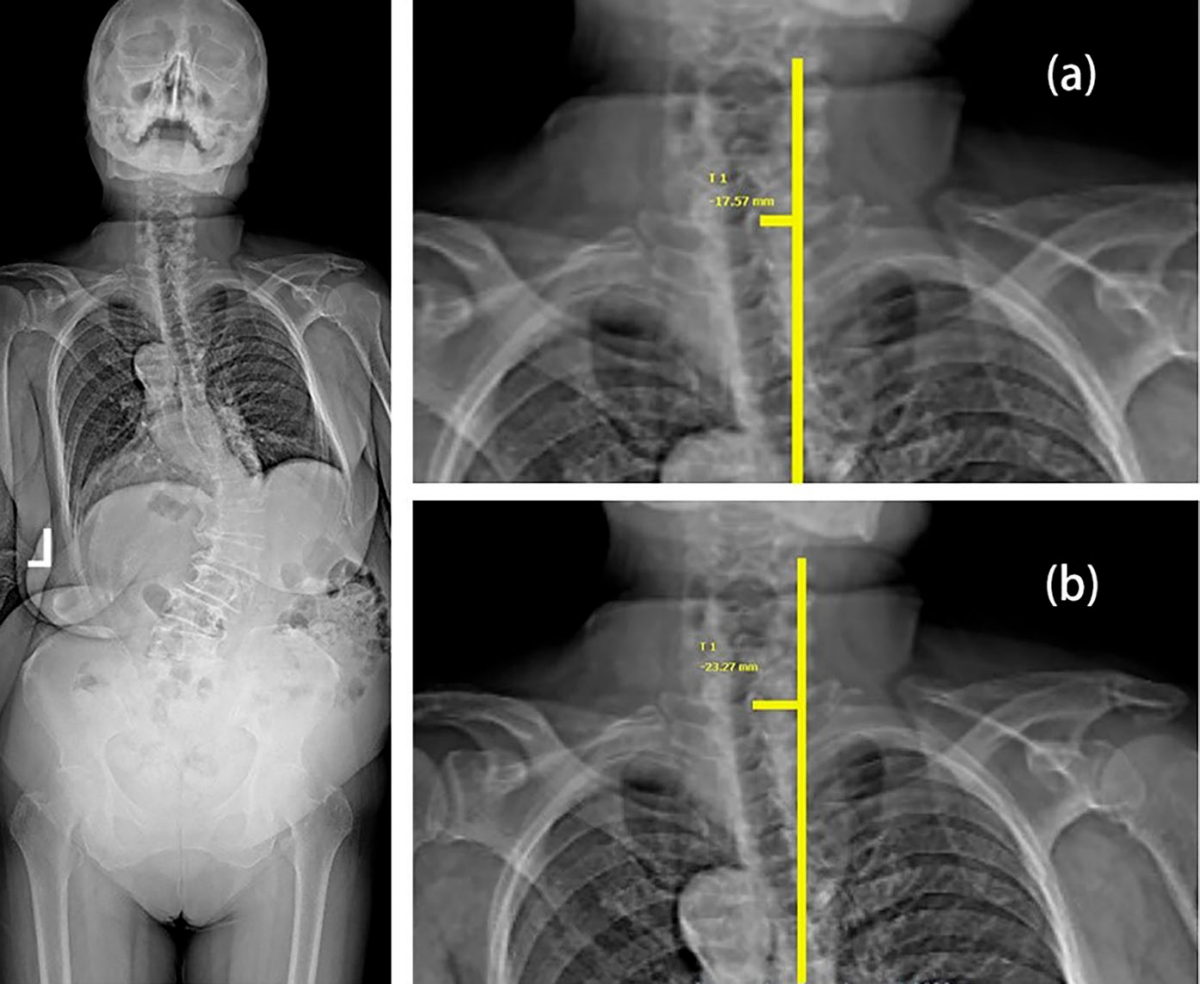
The measurement results of an observer. (a) The first measurement by the observer indicates a horizontal distance of 17.57 mm between T1PL and the midline of the pelvis. (b) The second measurement by the observer indicates a horizontal distance of 23.27 mm between T1PL and the midline of the pelvis.

Our baseline radiographic data indicated that a significant proportion of patients had coronal imbalance distances close to 2 cm, the threshold used in Obeid's classification. This might have contributed to the observed lower inter‐observer agreement, as small deviations in measurement can easily alter the classification from non‐CM to CM. In contrast, Qiu's system's 3 cm threshold provides a wider margin for measurement error, resulting in relatively higher inter‐observer agreement. For example, as shown in Figure 7, an initial measurement of the horizontal distance between T1PL and the midline of the pelvis in one case was 17.57 mm, leading the observer to classify the case as non‐CM. However, a re‐measurement 4 weeks later yielded 23.27 mm, surpassing Obeid's 2 cm threshold, and the observer reclassified the case as CM.

This example highlights the sensitivity of Obeid's classification system to minor variations, especially when distances are near the threshold. Additionally, factors such as patient positioning, image quality, and the complexity of spinal deformities can further affect measurement consistency. Slight changes in posture during X‐ray imaging or the presence of skeletal deformities can alter the position of landmarks like C7PL and T1PL, resulting in measurement discrepancies. Therefore, despite standardized training, the subjective nature of landmark identification remains a challenge, particularly when baseline measurements are close to the classification thresholds.

Consequently, we believe that the discrepancies observed between the two classification systems' inter‐ and intra‐rater agreement are largely due to these variations in baseline radiographic measurements and the inherent sensitivity of each system's threshold criteria. Further efforts to standardize measurement protocols and account for baseline variability are necessary to improve classification consistency.

### 4.5 Variability in Subtype Classification Within Obeid's System

**FIGURE 8:**
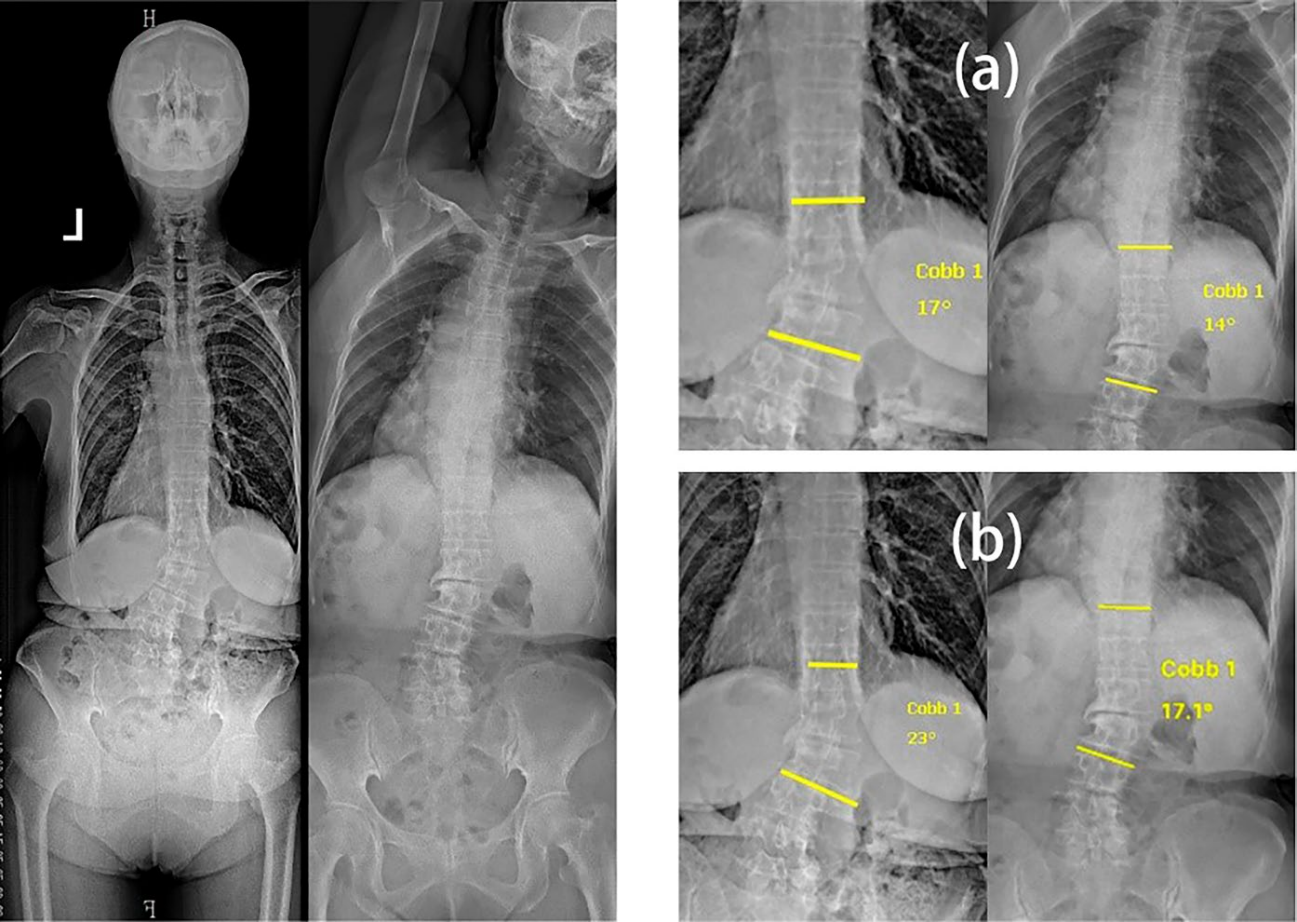
The measurement results of an observer. (a) Observer measurements: Standing Cobb angle of 17°, Cobb angle in the bending image of 14°. (b) Observer measurements: Standing Cobb angle of 23°, Cobb angle in the bending image of 17.1°.

In the process of inter‐ and intra‐rater agreement for the subtypes in Obeid's classification system, apart from the discrepancies mentioned earlier regarding whether to classify a case as CM, the main disagreements arise in the classification of T1A1, T1A2, T2A1, and T2A2 according to Obeid's classification system. We believe that this situation mainly stems from the subjective nature of each physician's judgment regarding the flexibility of the primary curve of the spine, the determination of degenerative changes in the spine, and other factors. As a result, inconsistencies in the classification of cases have emerged. In our experiment, the observer's assessment of the flexibility of the main curvature of spinal scoliosis in the case is determined using the flexibility of the structural curve (FSC) index. The FSC is calculated as the difference between the standing Cobb angle and the bending Cobb angle, divided by the standing Cobb angle. If the FSC is less than 0.25, the flexibility of the spine is considered poor. In this study, there are also variations in the measurements of the standing Cobb angle and the bending Cobb angle among different observers. Consequently, different observers may have varying assessments of spinal flexibility. The same observer might also provide different assessments at different times. As illustrated in Figure 8, slight deviations emerged in the measurements of the standing Cobb angle and the bending Cobb angle taken by two observers. Consequently, these deviations led to variations in the observers' assessments of spinal flexibility.

Even in cases of the aforementioned classification inconsistencies, both Qiu's classification system and Obeid's classification system demonstrated good performance in inter‐ and intra‐rater agreement. In Qiu's classification system, there was a substantial inter‐rater agreement when evaluating the classification categories (Type A, Type B, and Type C), and the intra‐rater agreement was nearly perfect. In Obeid's classification system, the inter‐rater agreement was almost perfect when evaluating the main classification categories (Type 0, Type 1, and Type 2), and the intra‐rater agreement was also almost perfect. When analyzing the complete classification system, including both types and subtypes, the inter‐rater agreement was substantial, and the intra‐rater agreement was also substantial.

### 4.6 Clinical Applications and Recommendations for Classification System Usage

Although both systems demonstrated strong reliability, they are designed to serve different clinical purposes. Qiu's classification system, with its simpler structure, is primarily focused on distinguishing between different CM patterns based on the direction and location of the primary scoliotic curve. This makes it particularly well‐suited for straightforward cases of degenerative scoliosis or adult scoliosis with minimal sagittal plane deformities, where a quick and clear classification is needed. Its use of C7PL and CSVL as primary landmarks allows for a less complicated classification process that can be easily implemented in routine clinical practice.

On the other hand, Obeid's classification system is more complex and provides a finer level of detail by considering factors such as curve mobility, degeneration at the lumbosacral junction, and coronal mobility of the lumbosacral junction. This system is especially useful in complex spinal deformities that involve both coronal and sagittal plane abnormalities or in cases where multiple deformities coexist. Because of its detailed subcategories and multiple classification criteria, Obeid's system is more applicable in specialized clinical settings where comprehensive assessment and treatment planning are required.

Overall, the choice of classification system should depend on the specific clinical scenario. Qiu's system may be more suitable for general clinical use and quick assessment, whereas Obeid's system is more appropriate for detailed evaluation and complex deformity management in specialized centers. Future research should aim to validate these classification systems in a wider range of clinical scenarios to better delineate their strengths and limitations.

To enhance consistency in using these two classification systems, we recommend that doctors take the following measures:

1. Clearly define standards: Ensure that the standards and requirements of the CM classification system are clearly defined and widely communicated within the team and organization. This ensures a shared understanding of the CM classification system and provides a common reference framework for evaluation.

2. Establish detailed processes: Develop detailed classification procedures that include specific steps, methods, and tools. This will help team members classify according to unified standards, reducing subjectivity, and individual differences.

3. Training and education: Provide training and educational opportunities to familiarize team members with the purpose, methods, and skills of the CM classification system. Training may include demonstrations, case studies, and simulated exercises to help them acquire the correct classification skills.

4. Establish a feedback mechanism: Establish an effective feedback mechanism that encourages team members to provide feedback and suggestions regarding CM classification. This aids in continuous improvement of the classification process and addresses any potential issues or difficulties.

5. Regular review and evaluation: Conduct periodic reviews and evaluations of the classification outcomes and effectiveness. By reviewing the classification results and gathering feedback from team members, potential issues can be identified and timely measures can be taken for improvement.

In summary, by clearly defining standards, establishing detailed processes, providing training and education, establishing a feedback mechanism and conducting regular reviews, the consistency of the CM classification system can be improved, ensuring the accuracy and reliability of the results.

### 4.7 Limitations and Strengths

Our study has several notable strengths. First, it is among the few studies that evaluate both inter‐ and intra‐observer agreement across two distinct classification systems for CM in ASD, offering valuable insights into their reliability and applicability in clinical settings. Second, the participation of experienced spine surgeons who received rigorous training in classification techniques strengthens the robustness and reproducibility of our findings. Finally, our large and diverse patient sample, drawn from multiple institutions, enhances the generalizability of the results, making them more applicable to a broader clinical population.

However, the limitations of our study include a certain bias in sample selection. All cases of spinal deformities in this study were from the First Affiliated Hospital of Xinjiang Medical University and Xinhua Hospital Affiliated to Shanghai Jiao Tong University. If the cases are only from specific regions or specific populations, the results may not be representative of the entire population's prevalence of the disease. Although the selection of cases is unrelated to the subsequent classification of cases by doctors, prior to the start of this study, we estimated the required sample size, which involved data on the prevalence of different types of spinal deformities. If the prevalence of our cases differs to some extent from the prevalence in the entire population, it could result in a discrepancy between the estimated sample size and the actual sample size required, thus affecting our research results. Moreover, the treatment strategies and clinical practices at these two hospitals may differ from those in other regions or medical centers, potentially affecting the observed inter‐ and intra‐rater agreement. This could lead to biases in the assessment of the classification systems' reliability, as different clinical settings may influence the interpretation of radiographic images or the emphasis on certain anatomical landmarks. Consequently, the generalizability of our findings to other populations or healthcare systems may be limited. We acknowledge that the restricted sample selection may reduce the external validity of our findings, and future studies should aim to include a broader range of clinical settings and patient demographics to further validate the classification systems.

To overcome sample selection bias, future researchers can employ specific strategies. For instance, when selecting cases, ensuring diversity in the sample, including factors such as age, gender, cultural background, region, hospital, and ethnicity, will help ensure that the sample represents the entire population. Additionally, future studies should consider conducting multi‐center research to validate the classification systems across diverse clinical settings. This approach can help identify whether the observed classification inconsistencies are due to inherent limitations of the systems or are influenced by local clinical practices. Furthermore, future research could explore refining the classification thresholds and incorporating additional imaging techniques, such as 3D imaging or dynamic radiographs, to enhance the accuracy and reliability of these classification systems in more complex cases.

## 5 Conclusion

This study carefully examined the inter‐ and intra‐observer agreement of the coronal imbalance classification systems proposed by Qiu and Obeid, yielding important insights. The investigation revealed significant consensus among different medical experts and even within the same expert across various evaluations, particularly with nearly perfect agreement found in Obeid's classification system. These results indicate that these classification systems provide a solid foundation for the categorization and diagnosis of spinal coronal imbalance. The study confirms that, regardless of varying contexts, medical professionals can consistently and accurately classify and diagnose these conditions. This research not only has significant implications for clinical practice but also offers valuable lessons for future studies and treatment methods. By enabling more precise identification and categorization of spinal coronal imbalance, it holds the potential to enhance patient quality of life and optimize treatment outcomes. This study lays a robust groundwork for medical advancement and the maintenance of spinal health.

## Author Contributions

Mu.M. and Ma.M. contributed equally to this work and are co‐first authors. Mu.M. drafted the manuscript and conducted the primary data analysis. Ma.M. designed the study and participated in data collection. P.Y., X.‐Y.C., G.‐J.F., and T.X. assisted with data interpretation and contributed to the statistical analysis. B.L., P.‐B.C., and Z.‐Y.L. reviewed the manuscript and provided critical revisions. W.‐B.S., S.‐D.J., and L.‐S.J. supervised the research process and ensured the study's integrity. X.‐F.Z. coordinated the research activities, reviewed the final manuscript, and approved it for submission. All authors reviewed and approved the final manuscript.

## Conflicts of Interest

The authors declare no conflicts of interest.

## Supporting information


File S1.

File S2.
