# Supplementary material for: An Inter‐ and Intra‐Rater Agreement Assessment of Two Novel Classification Systems for Coronal Imbalance in Adult Scoliosis and Spine Deformity
Source: Orthop Surg. 2025 Jan 20;17(4):1075–85. doi: 10.1111/os.14356 (PMC11962280; doi:10.1111/os.14356)
Supplement: Supplementary file 2 — File S2. [file OS-17-1075-s002.docx]

**Table 4 Summary of Classification Outcomes by Qiu and Obeid Systems**

| **Doctor / Classification** | **Qiu (First Round)**  **N (%)** | **Obeid (First Round)**  **N (%)** | **Qiu (Second Round)**  **N (%)** | **Obeid (Second Round)**  **N (%)** |
| --- | --- | --- | --- | --- |
| ***Doctor 1*** |  |  |  |  |
| A: | 36 (51.4%) | / | 34 (48.6%) | / |
| B: | 16 (22.9%) | / | 18 (25.7%) | / |
| C: | 18 (25.7%) | / | 18 (25.7%) | / |
| 0: | / | 29 (41.4%) | / | 24 (34.3%) |
| 1A1: | / | 7 (10.0%) | / | 13 (18.6%) |
| 1A2: | / | 10 (14.3%) | / | 6 (8.6%) |
| 1B: | / | 4 (5.7%) | / | 3 (4.3%) |
| 2A1: | / | 14 (20.0%) | / | 16 (22.9%) |
| 2A2: | / | 5 (7.1%) | / | 6 (8.6%) |
| 2B | / | 1 (1.4%) | / | 2 (2.9%) |
| ***Doctor 2*** |  |  |  |  |
| A: | 32 (45.7%) | / | 30 (42.9%) | / |
| B: | 20 (28.6%) | / | 20 (28.6%) | / |
| C: | 18 (25.7%) | / | 20 (28.6%) | / |
| 0: | / | 29 (41.4%) | / | 23 (32.9%) |
| 1A1: | / | 9 (12.9%) | / | 14 (20.0%) |
| 1A2: | / | 7 (10.0%) | / | 6 (8.6%) |
| 1B: | / | 3 (4.3%) | / | 3 (4.3%) |
| 2A1: | / | 16 (22.9%) | / | 13 (18.6%) |
| 2A2: | / | 5 (7.1%) | / | 9 (12.9%) |
| 2B | / | 1 (1.4%) | / | 2 (2.9%) |
| ***Doctor 3*** |  |  |  |  |
| A: | 34 (48.6%) | / | 31 (44.3%) | / |
| B: | 18 (25.7%) | / | 19 (27.1%) | / |
| C: | 18 (25.7%) | / | 20 (28.6%) | / |
| 0: | / | 26 (37.1%) | / | 27 (38.6%) |
| 1A1: | / | 7 (10.0%) | / | 7 (10.0%) |
| 1A2: | / | 11 (15.7%) | / | 11 (15.7%) |
| 1B: | / | 3 (4.3%) | / | 4 (5.7%) |
| 2A1: | / | 15 (21.4%) | / | 15 (21.4%) |
| 2A2: | / | 5 (7.1%) | / | 5 (7.1%) |
| 2B | / | 3 (4.3%) | / | 1 (1.4%) |
| ***Doctor 4*** |  |  |  |  |
| A: | 28 (40.0%) | / | 31 (44.3%) | / |
| B: | 21 (30.0%) | / | 21 (30.0%) | / |
| C: | 21 (30.0%) | / | 18 (25.7%) | / |
| 0: | / | 27 (38.6%) | / | 25 (35.7%) |
| 1A1: | / | 10 (14.3%) | / | 12 (17.1%) |
| 1A2: | / | 7 (10.0%) | / | 7 (10.0%) |
| 1B: | / | 3 (4.3%) | / | 3 (4.3%) |
| 2A1: | / | 18 (25.7%) | / | 16 (22.9%) |
| 2A2: | / | 4 (5.7%) | / | 5 (7.1%) |
| 2B | / | 1 (1.4%) | / | 2 (2.9%) |
| ***Doctor 5*** |  |  |  |  |
| A: | 33 (47.1%) | / | 32 (45.7%) | / |
| B: | 19 (27.1%) | / | 18 (25.7%) | / |
| C: | 18 (25.7%) | / | 20 (28.6%) | / |
| 0: | / | 29 (41.4%) | / | 27 (38.6%) |
| 1A1: | / | 10 (14.3%) | / | 8 (11.4%) |
| 1A2: | / | 7 (10.0%) | / | 9 (12.9%) |
| 1B: | / | 3 (4.3%) | / | 3 (4.3%) |
| 2A1: | / | 13 (18.6%) | / | 14 (20.0%) |
| 2A2: | / | 7 (10.0%) | / | 7 (10.0%) |
| 2B | / | 1 (1.4%) | / | 2 (2.9%) |
| ***Doctor 6*** |  |  |  |  |
| A: | 34 (48.6%) | / | 36 (51.4%) | / |
| B: | 16 (22.9%) | / | 16 (22.9%) | / |
| C: | 20 (28.6%) | / | 18 (25.7%) | / |
| 0: | / | 27 (38.6%) | / | 25 (35.7%) |
| 1A1: | / | 8 (11.4%) | / | 10 (14.3%) |
| 1A2: | / | 9 (12.9%) | / | 8 (11.4%) |
| 1B: | / | 3 (4.3%) | / | 3 (4.3%) |
| 2A1: | / | 14 (20.0%) | / | 16 (22.9%) |
| 2A2: | / | 7 (10.0%) | / | 6 (8.6%) |
| 2B | / | 2 (2.9%) | / | 2 (2.9%) |
